# Climate-driven infectious disease risks: a global scoping review of epidemiological patterns, methodological gaps, and policy imperatives

**DOI:** 10.1186/s12879-025-12214-5

**Published:** 2025-12-29

**Authors:** İzzet Çeleğen, Abdullah Sarıöz

**Affiliations:** Department of Public Health, Faculty of Medicine, Yuzuncu Yil University, Van, 65080 Türkiye

**Keywords:** Climate change, Infectious diseases, Scoping review, Public health policy, Thematic analysis

## Abstract

**Background:**

Climate change is reshaping infectious disease dynamics worldwide, yet existing evidence remains fragmented, geographically imbalanced, and weakly connected to policy.

**Objective:**

To map global peer-reviewed literature (2010–2025) on climate-related infectious diseases, identify epidemiological patterns, methodological gaps, and policy relevance.

**Methods:**

Following PRISMA-ScR and Joanna Briggs guidance, PubMed, Web of Science, and Scopus were searched for English-language studies (January 2010–January 2025). Of 2,239 screened records, 2,139 met inclusion criteria. Ten predefined variables were extracted and thematically coded using Python-assisted and manual validation.

**Results:**

Observational designs constituted the largest share (38.9%), followed by modeling studies (34.8%). Respiratory (36.2%) and vector-borne (34.9%) infections were the most frequent topics. Composite climate indicators were used in 29.8% of studies. Only 27% contained explicit policy recommendations, and spatial modeling appeared in 13%. Early warning systems were mentioned in 57.5% (*n* = 1,230) of articles, whereas socioeconomic determinants were included in only 13.4%, mostly from high-income settings. Low-income and climate-vulnerable regions remained markedly underrepresented.

**Conclusions:**

Despite rapid expansion of climate-health research over the past 15 years, key deficits persist in methodological diversity, policy translation, and regional equity. Priorities for the next phase include longitudinal designs, integration of socioeconomic indicators, and harmonized One Health frameworks linking surveillance and adaptation. Strengthening these dimensions is essential for shifting global preparedness for climate-sensitive infectious diseases from reactive to preventive.

**Clinical trial registration:**

Not applicable.

**Supplementary Information:**

The online version contains supplementary material available at 10.1186/s12879-025-12214-5.

## Background

Climate change increasingly dominates the global health agenda by altering the spatiotemporal patterns of infectious diseases. A growing body of global evidence shows that rising temperatures, precipitation variability, and ecosystem disruption are reshaping disease dynamics worldwide [1–3]. Recent syntheses indicate that over 80% of reviewed studies identify positive associations between climate variability and infectious disease transmission, particularly for vector-borne and waterborne pathogens [1]. Cumulative reviews confirm consistent mechanistic pathways but highlight fragmentation in methodological design, as single-factor models still dominate the literature and rarely integrate socioeconomic or ecological determinants [2, 3].

This fragmentation extends to both disease focus and geography. The existing evidence base is heavily concentrated on malaria and dengue, while neglected tropical and zoonotic diseases remain understudied [4]. Only a minority of climate–health studies address surveillance, adaptation, or policy implications, and cross-sectoral frameworks such as One Health are seldom operationalized [5, 6]. Regional analyses further reveal how shifting climate conditions alter vector distributions in tropical ecosystems [7] and how evidence gaps persist in sub-Saharan Africa regarding monitoring and policy integration [8]. Even in temperate regions, extreme rainfall and flooding have been linked with outbreaks of waterborne infections, underscoring the universality of climate sensitivity [9]. Health system resilience and governance capacity also emerge as critical determinants of actual disease burden, particularly in low-income settings [10].

At the national and subnational scales, predictive modeling demonstrates how climate projections can inform early warning systems and guide adaptive responses [11, 12]. Comparative analyses of surveillance systems across Europe and other regions reveal persistent gaps in standardization and preparedness [13]. Beyond technical limitations, climate vulnerability is increasingly understood as intertwined with socioeconomic inequality, reinforcing the need for integrative approaches that link health, environment, and social policy [14, 15].

This scoping review directly addresses these deficiencies. It provides a comprehensive synthesis of peer-reviewed evidence published between 2010 and 2025, capturing the post-pandemic evolution of climate–infectious-disease research. The review applies a cross-regional equity lens to assess how evidence production varies by geography and income level, and it quantitatively evaluates how policy and early warning content are embedded within the literature. Through this integrative design, the study aims to clarify where global evidence is concentrated, where critical gaps remain, and how future research can better support equitable, data-driven, and adaptive public health strategies.

## Methods

This scoping review was conducted in accordance with the PRISMA Extension for Scoping Reviews (PRISMA-ScR) guidelines [16] and the methodological recommendations of the Joanna Briggs Institute [17]. The review aimed to systematically map and synthesize peer-reviewed scientific literature published between January 1, 2010, and January 1, 2025, exploring the association between climate change and the epidemiology of infectious diseases, alongside identifying public health responses, surveillance strategies, and climate-adaptive policy approaches.

A comprehensive literature search was carried out between June 1 and June 15, 2025, using three major electronic databases: PubMed, Web of Science (WoS), and Scopus. The search strategy combined Boolean operators, free-text terms, and controlled vocabulary (MeSH terms for PubMed, Emtree for Scopus), incorporating keywords such as “climate change,” “infectious disease,” “epidemiology,” “surveillance,” and “public health adaptation.” Detailed search strategies and database-specific queries are provided in Supplementary Table 1. Only English-language articles published between 2010 and 2025 were considered eligible, and grey literature sources were not included.

**Fig. 1:**
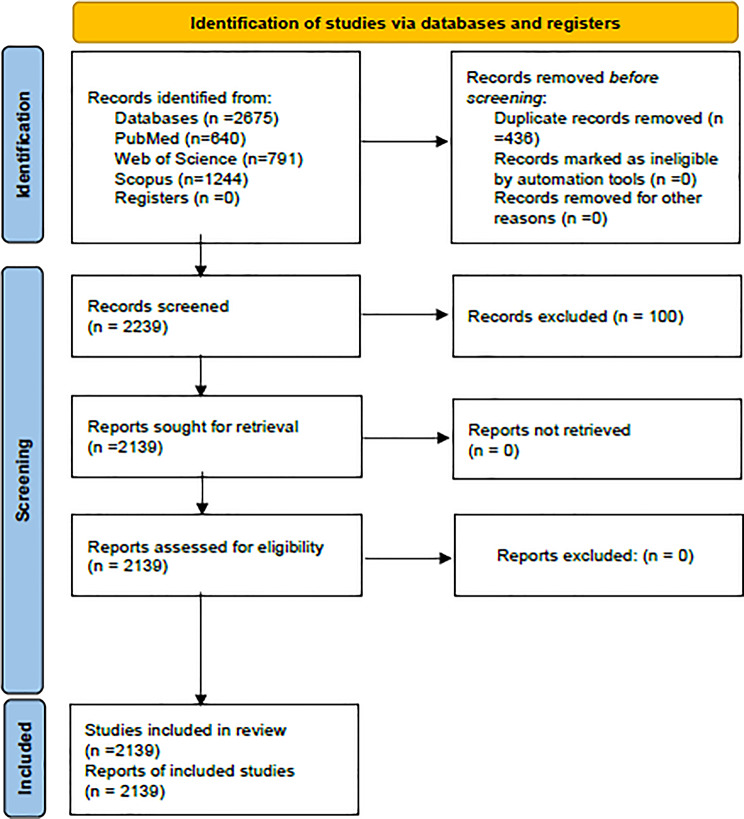
Study selection flow (PRISMA-ScR, based on PRISMA 2020)

The initial search yielded 2,675 records. After removing 436 duplicates using Python (version 3.11) with pandas (version 2.0) and manual verification, 2,239 unique records were retained for screening. Titles and abstracts were independently reviewed by two researchers using Microsoft Excel, followed by full-text assessment where necessary. Discrepancies were resolved through consensus discussions. No automation tools were used for study selection or eligibility screening; all steps were conducted manually to ensure methodological consistency. The study selection process is detailed in Fig. 1, following the PRISMA 2020 flow diagram guidelines [16]. Automation was exclusively applied in data extraction and thematic coding phases, as described below. Inter-rater agreement was assessed using Cohen’s kappa coefficient, calculated as κ = 0.84, indicating substantial agreement.

The inclusion criteria comprised English-language studies published within the defined time frame that addressed the impact of climate-related factors — including temperature rise, precipitation variability, extreme weather events, and natural disasters — on infectious disease occurrence, distribution, or dynamics, and/or studies reporting public health responses such as surveillance systems, adaptation measures, or policy-level interventions. Studies that were animal-only experimental research, clinical studies not linking infection with climate exposure, or non-research publications such as commentaries, editorials, letters, or abstracts without full text were excluded.

Data extraction was performed using a structured framework in Microsoft Excel 365. Ten predefined variables were systematically recorded: publication year, country or region of study, study design, climate factor, disease type, affected population, public health intervention type, study outcome, methodological approach, and key findings. The complete thematic classification output, including all extracted variables and coding structures applied during the review, is available as Supplementary Data Set 2.

Study designs were categorized at two hierarchical levels to maintain internal consistency across the review. At the first level, studies were broadly grouped as observational, experimental, modeling/simulation, or review/meta-analysis. At the second level, observational studies were further disaggregated into cross-sectional, case-control, cohort, and ecological designs. Modeling studies encompassed both statistical and computational projection models. This two-tiered structure ensured alignment between quantitative mapping and thematic coding, allowing for accurate comparison of methodological trends across regions and disease categories.

To ensure comparability and interpretability across the diverse body of evidence, exposure and outcome variables were harmonized prior to coding. Climatic exposures were standardized into three categories: (i) temperature-related factors (mean, maximum, heatwaves), (ii) precipitation and hydrometeorological factors (rainfall, humidity, flooding), and (iii) extreme weather events and disasters. Disease outcomes were coded into four major classes: vector-borne, waterborne, respiratory, and zoonotic infections. When studies reported composite indicators (e.g., “climate variability index” or “multi-disease surveillance outcomes”), these were decomposed and assigned to the most relevant category based on the primary analytical focus. This harmonization process reduced heterogeneity and facilitated cross-study comparison. This classification framework was developed a priori based on the most commonly used exposure and outcome constructs in climate–health literature, and all comparative analyses in this review were anchored to these standardized categories.

Following data extraction, climate exposures and disease types were categorized into broader thematic groups: climatic drivers (temperature, precipitation, disasters), infectious disease categories (vector-borne, waterborne, respiratory, zoonotic), and public health strategies (surveillance, adaptation, early warning systems). Data standardization and thematic coding were conducted using Python (version 3.11), applying pandas (version 2.0) and spaCy (version 3.6) libraries. Coding was guided by six core domains identified a priori through literature review and consultation with two independent public health experts specialized in climate-health research, ensuring content validity. A rule-based keyword algorithm, supplemented by manual validation, was employed to ensure consistency in thematic classification. Python scripts and keyword lists used for this process are available upon reasonable request.

To complement thematic mapping, we also quantified publication concentration using a country-level Gini index, computed on annual publication counts (Python 3.11; pandas library). Values close to 0 indicate perfect equality and those near 1 indicate maximum concentration. This metric was used to evaluate geographic inequality in research output across countries and income groups.

Of the 2,239 screened studies, 2,139 were ultimately included in the review. The difference was due to the exclusion of articles without full-text availability or those deemed irrelevant upon full-text evaluation.

### Ethical approval

was not required for this scoping review, as all analyzed data were derived from publicly available peer-reviewed literature without involving human participants or identifiable personal information.

## Results

### General characteristics of included studies

**Fig. 2:**
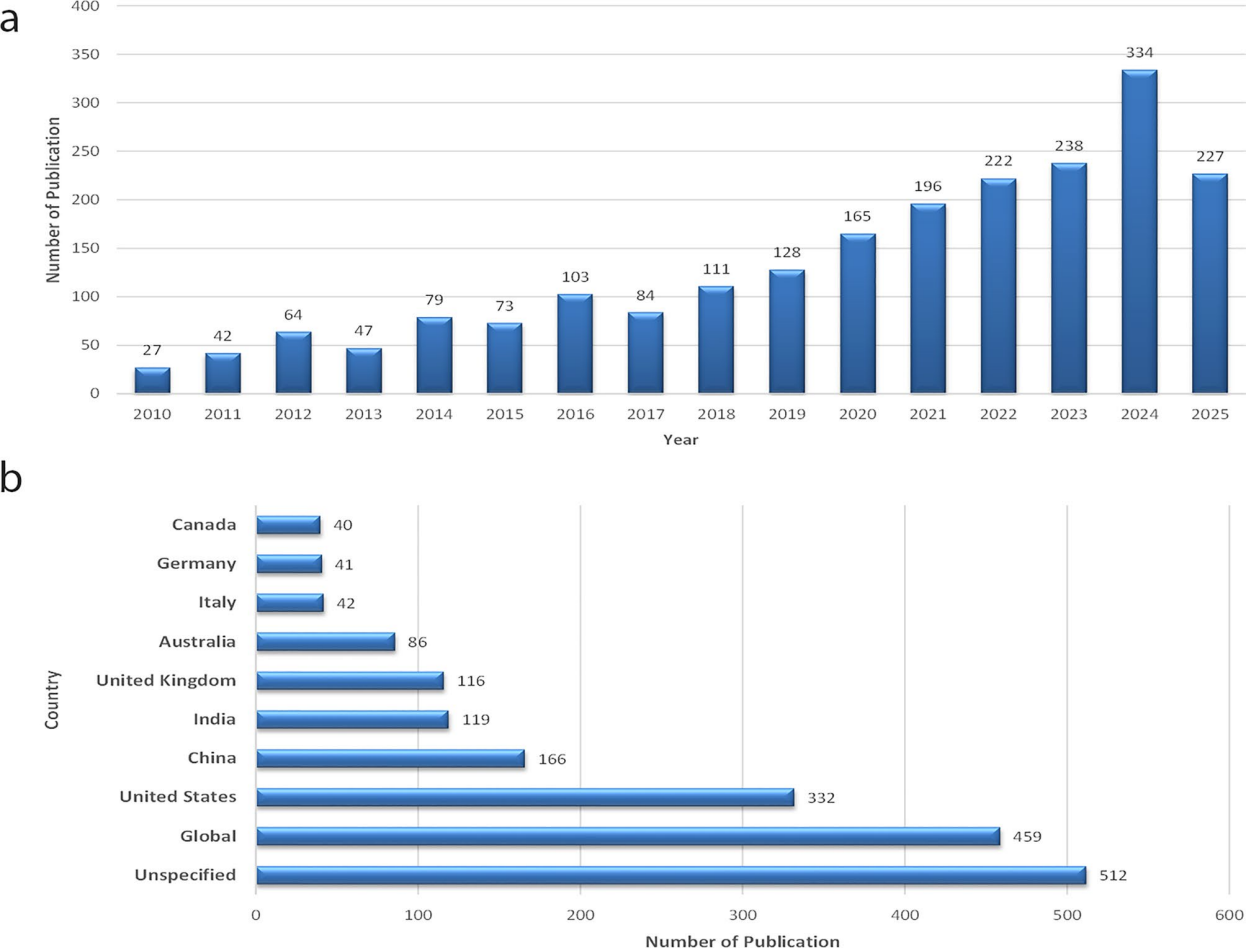
**a–b.** (**a**) Publications by Year (2010–2025); (**b**) Top 10 Countries by Publication Count

A total of 2,139 studies were included in this scoping review. The annual number of publications showed a steady rise from 2010 to 2024, peaking in 2024 (*n* = 334, 15.6%) (Fig. 2a–b).

The United States (*n* = 332, 15.5%), China (*n* = 166, 7.8%), and India (*n* = 119, 5.6%) were the most prolific contributors, followed by the United Kingdom, Australia, and Brazil.

When analyzed by WHO region, most studies originated from the Region of the Americas (33.8%) and the Western Pacific (26.4%), whereas the African (8.1%) and Eastern Mediterranean (6.7%) regions were less represented. Only 4.3% of studies were from low-income countries, compared to 71.2% from high- and upper-middle-income economies.

**Fig. 3:**
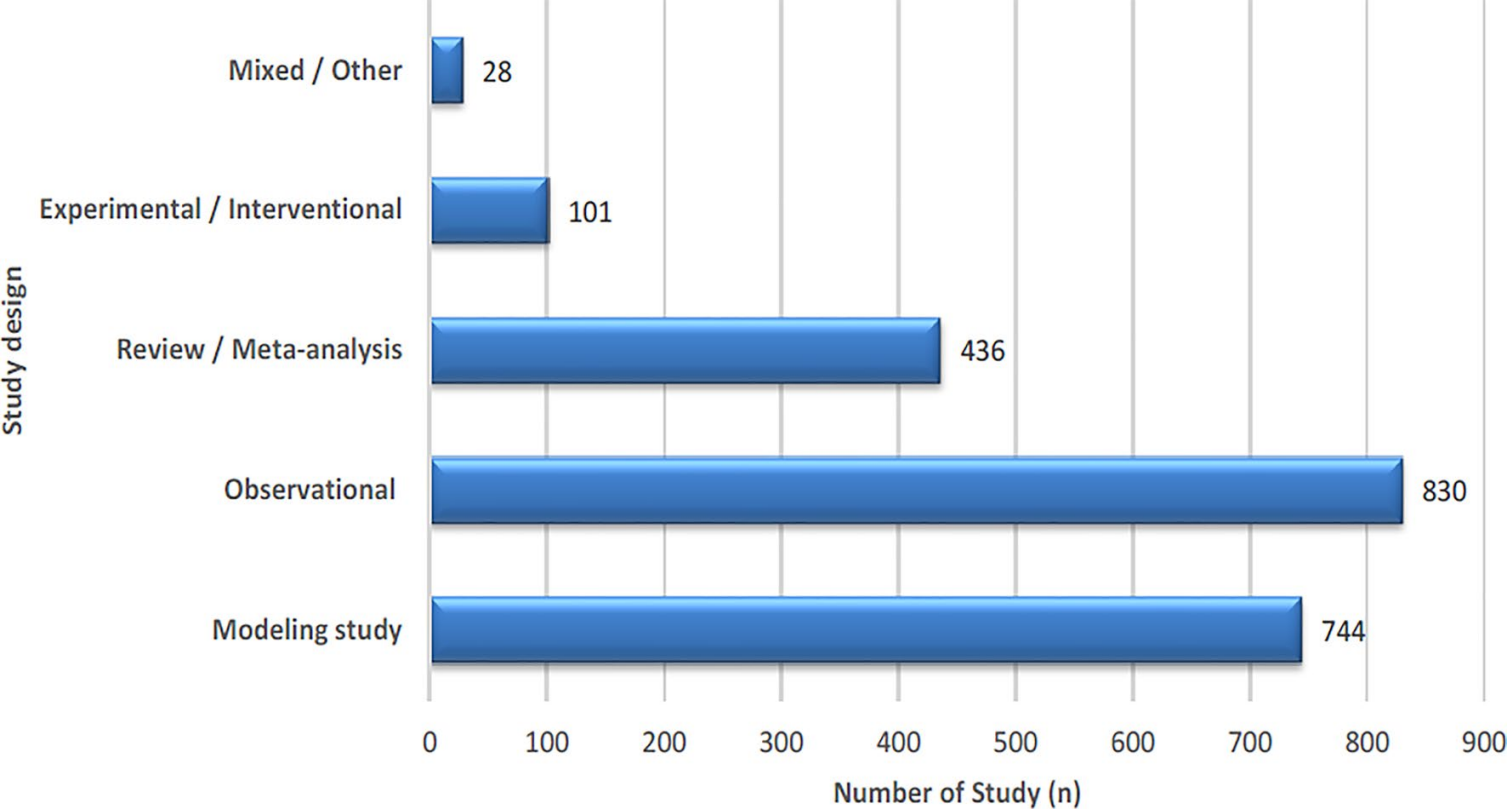
Distribution of study designs in the included studies (*n* = 2,139)

Modeling and simulation approaches accounted for 34.8% of all included studies (*n* = 744). Observational designs constituted the largest group (*n* = 830, 38.9%), encompassing cross-sectional, case–control, cohort, and ecological studies. Review and meta-analytic studies represented 20.4% (*n* = 436), and experimental or interventional designs comprised 4.7% (*n* = 101). A small fraction (*n* = 28, 1.3%) were classified as mixed or other, including qualitative and policy-oriented analyses (Fig. 3).

Study designs were categorized at two hierarchical levels to ensure internal consistency: (1) broad methodological groups—observational, experimental/interventional, modeling/simulation, and review/meta-analysis; and (2) subtypes within observational studies (cross-sectional, case-control, cohort, ecological).

### Climatic factors associated with health outcomes

Across the included studies, climatic drivers were predominantly represented by composite climate indices (*n* = 637, 29.8%), such as combined measures of temperature, humidity, and precipitation variability.

Specific single-variable exposures, including temperature rise (*n* = 143, 6.7%) and precipitation anomalies (*n* = 101, 4.7%), were less frequent.

### Disease types and regional patterns

Respiratory infections (*n* = 775, 36.2%) and vector-borne diseases (*n* = 746, 34.9%) were the most frequently studied categories among the included articles (Table 1).

When stratified by region, vector-borne diseases were most common in Africa (8.4%) and Asia (7.6%), while respiratory infections were most frequent in Europe (15.9%) and the Americas (10.7%).

Water and foodborne infections represented a smaller proportion of studies, with most work originating from Asia and Europe.

Zoonotic diseases were distributed relatively evenly across regions, whereas Oceania contributed fewer studies (≤ 3% across all categories).

At the global level, the distribution of publications was highly unequal across countries (concentration/Gini index = 0.76), indicating a strong clustering of research output within high-income regions.

At the country level, the United States (*n* = 332, 15.5%), China (*n* = 166, 7.8%), and India (*n* = 119, 5.6%) were the leading contributors, accounting together for approximately 29% of all publications.

Although Africa and Asia are not individually displayed in the earlier country-level summary, the *‘Global / Multi-country’* category (*n* = 341, 15.9%) primarily includes multicenter or systematic reviews that combine data from African (56%) and Asian (29%) contexts.

**Table 1:** Disease types and regional distribution with percentages

Region	Respiratory (*n*, %)	Vector-borne (*n*, %)	Water/Foodborne (*n*, %)	Zoonotic (*n*, %)	Other / Unspecified (*n*, %)
Africa	124 (5.8%)	180 (8.4%)	27 (1.3%)	19 (0.9%)	20 (0.9%)
Asia	158 (7.4%)	162 (7.6%)	35 (1.6%)	22 (1.0%)	26 (1.2%)
Europe	341 (15.9%)	90 (4.2%)	48 (2.2%)	25 (1.2%)	30 (1.4%)
Americas	230 (10.7%)	63 (2.9%)	21 (1.0%)	14 (0.7%)	18 (0.8%)
Oceania	49 (2.3%)	18 (0.8%)	3 (0.1%)	5 (0.2%)	7 (0.3%)
Global / Multi-country	341 (15.9%)	338 (15.8%)	64 (3.0%)	47 (2.2%)	181 (8.5%)

### Public health interventions

Surveillance and early warning systems were examined in 1,230 studies (57.5%), while adaptation policies were addressed in 156 studies (7.3%). Here, monitoring/surveillance refers to routine data systems; early warning systems (EWS) denote predictive/threshold-based alert architectures.

Policy-related publications were most frequently derived from global or high-income contexts.

### Methodological trends and gaps

Modeling studies were common across the included literature, with spatial modeling techniques reported in 278 studies (13.0%), primarily focused on vector-borne diseases.

When stratified by income level, complex study designs—such as longitudinal, cohort, and interventional approaches—were most frequently conducted in high-income settings, whereas such designs were less frequent in low-income regions.

### Thematic gaps and opportunities

Based on thematic coding results (Table 2), 27% of the included studies explicitly reported structured policy recommendations. Early warning systems and infrastructure capacity were referenced in 1,230 and 793 studies, respectively, but most did not include detailed implementation frameworks. Socioeconomic factors were incorporated in fewer than 15% of studies.

**Table 2:** Thematic coding frequency and gap analysis*

Theme	Number of Studies (*n*, %)	Identified Gap
Policies and Strategies	579 (27.1%)	Structured policy frameworks rarely provided
Modeling/Spatial Modeling	359 (16.8%)	Mainly focused on vector-borne diseases
Monitoring Systems	861 (40.3%)	Detailed implementation lacking
Socioeconomic Factors	286 (13.4%)	Limited to correlational analyses
Infrastructure and Capacity	793 (37.1%)	Detailed implementation lacking

*Percentages may not sum to 100% due to multi−categorization and overlapping classifications

## Discussion

### Summary of key findings

This scoping review synthesized 2,139 peer-reviewed studies published between 2010 and 2025 examining the relationship between climate change and infectious disease dynamics. The analysis revealed a steady increase in publication volume—especially between 2020 and 2024—coinciding with the post-pandemic expansion of climate–health research. Modeling and observational designs predominated, while experimental and policy-focused investigations remained comparatively limited. Respiratory and vector-borne diseases were the most frequently studied categories, and most research originated from high- and upper-middle-income regions. Despite the growing global interest, representation from low-income countries and region-specific policy evaluations remains limited, echoing previously reported disparities in global health research [18, 19].

### Comparative interpretation and novel contributions

Unlike earlier reviews that concluded around 2022–2023 [20–23], this study extends the evidence base to 2025, capturing the post-pandemic evolution of climate–infectious-disease research. Two key developments are evident. First, there has been a gradual methodological shift toward predictive and spatial modeling, including initial applications of machine learning. Second, the thematic scope has broadened beyond vector-borne infections to increasingly include respiratory and zoonotic diseases linked to climatic variability. Earlier reviews mostly analyzed single climatic factors such as temperature or rainfall [24–26]. In contrast, the present synthesis demonstrates a growing use of composite climate indicators—integrating temperature, humidity, and precipitation variability—to capture complex exposure–outcome relationships. These trends align with broader movements in climate–health research emphasizing multidimensional and systems-based approaches [14, 27].

### Policy translation and actionable frameworks

Only 27% of studies explicitly reported policy content, confirming the persistence of a translation gap between scientific evidence and decision-making [15, 28].

Most publications emphasized surveillance and modeling, while evaluations of real-world adaptation or early warning programs were rare.

This imbalance suggests that evidence generation continues to outpace implementation capacity.

This policy gap manifests across three interrelated dimensions:

1. Implementation gap — Surveillance frameworks were conceptually robust but lacked integration with national systems.
2. Equity gap — Most policy-oriented research originated from high-income countries, leaving vulnerable regions underrepresented.
3. Integration gap — “One Health” and “Planetary Health” paradigms were frequently cited but seldom operationalized.

Emerging frameworks such as the WHO Climate Change and Health Strategy (2023–2030) and the Lancet Countdown indicators offer structured mechanisms for embedding climate intelligence into health planning, yet few studies have applied them empirically [29, 30].

Aligning evidence production with regional vulnerability through field validation, cost-effectiveness analyses, and cross-regional pilot programs will be essential to bridge this gap.

Operational uptake could be accelerated by aligning national plans to these frameworks’ indicator sets and reporting cycles.

Findings support integrated pipelines that combine projection modeling with longitudinal and operational data to improve early warning calibration and policy uptake.

### Spatial disparities and research equity

Geographical asymmetries observed in this dataset reflect structural inequities in global research capacity. Africa (8.1%) and the Eastern Mediterranean (6.7%) were notably underrepresented, mirroring disparities in research funding and data infrastructure. Such gaps, also emphasized by Ebi et al. [27] and the broader climate–inequality literature [15], constrain global situational awareness and preparedness for climate-sensitive epidemics. This pattern is quantitatively supported by a concentration index of 0.76, confirming the pronounced spatial clustering of global climate–health research within higher-income regions. Although spatial modeling has become conceptually central, only 278 of 2,139 studies (13.0%) applied it—mainly in vector-borne disease contexts—limiting early warning potential for respiratory and waterborne diseases. Expanding open-access geospatial repositories, standardized metadata protocols, and regional research partnerships could enhance analytic equity and representation.

### Methodological gaps and future directions

Modeling approaches dominate, but the scarcity of longitudinal and interventional designs limits causal inference. Few studies incorporated socioeconomic or infrastructural covariates, and less than 15% addressed equity or vulnerability dimensions. This omission hampers context-sensitive adaptation strategies, underscoring the need for integrative, implementation-oriented research [31].

### Future investigations should prioritize

- Integration of socioeconomic indicators into predictive frameworks;
- Longitudinal and interventional designs capturing lagged climate effects;
- Mixed-methods evaluation of adaptation and mitigation policies;
- Capacity-building and data infrastructure in underrepresented regions.

### Overall implications

Collectively, the findings indicate that climate–infectious-disease research is transitioning from exploratory modeling toward more operational and policy-relevant science. However, the imbalance between predictive and applied studies persists, highlighting the need for stronger feedback loops between evidence generation and policy action. The post-2020 literature confirms that climate change has become an active driver of infectious-disease dynamics, underscoring the urgent need for globally coordinated yet locally contextualized policies that strengthen surveillance, preparedness, and adaptive capacity.

### Limitations

This review has several limitations. First, potential inaccuracies in country attribution across original studies may have influenced regional analyses. Second, restricting the search to English-language and peer-reviewed literature excluded grey and non-English sources, possibly omitting regionally relevant findings. Third, methodological heterogeneity—from descriptive surveys to complex spatial models—posed challenges for synthesis. Finally, the exclusion of operational data from health systems may limit the practical applicability of some conclusions. These restrictions likely undercount region-specific evidence from francophone and lusophone Africa and parts of Latin America.

## Conclusion

This scoping review maps and synthesizes global evidence published between 2010 and 2025 on the intersection of climate change and infectious disease epidemiology. It delineates methodological trends, regional distributions, and thematic gaps across more than two thousand studies, providing a structured foundation for future analyses and policy frameworks addressing climate-sensitive infectious diseases.

## Supplementary Information

Below is the link to the electronic supplementary material.

Supplementary Material 1

Supplementary Material 2

## Data Availability

All data generated or analysed during this study are included in this published article and its supplementary information files.

## Declarations

### Use of AI tools

Generative AI tools (ChatGPT, OpenAI, 2025) were used only to improve language clarity. No AI tools were used for study screening, data extraction, coding, analysis, or interpretation. All analytical steps were performed manually or via rule-based Python scripts under author supervision.

## Abbreviations

BMC: BioMed Central
HICs: High-Income Countries
JBI: Joanna Briggs Institute
LMICs: Low- and Middle-Income Countries
PRISMA-ScR: Preferred Reporting Items for Systematic Reviews and Meta-Analyses extension for Scoping Reviews
One Health: Human–Animal–Environment Integrated Health Approach
WHO: World Health Organization
