# Supplementary material for: Climate-driven infectious disease risks: a global scoping review of epidemiological patterns, methodological gaps, and policy imperatives
Source: BMC Infect Dis. 2025 Dec 29;25:1770. doi: 10.1186/s12879-025-12214-5 (PMC12750609; doi:10.1186/s12879-025-12214-5)
Supplement: Supplementary file 1 — Supplementary Material 1 [file 12879_2025_12214_MOESM1_ESM.docx]

| **Supplementary Table 1. Detailed Search Strategies** | | | |
| --- | --- | --- | --- |
| **Database** | **Search String Example** | **Date of Search** | **Filters Applied** |
| **PubMed** | ("climate change"[MeSH Terms] OR "climate change"[All Fields] OR "global warming"[All Fields] OR "environmental change"[All Fields]) AND ("infectious disease"[MeSH Terms] OR "infectious disease"[All Fields] OR "epidemiology"[Subheading] OR "surveillance"[All Fields] OR "public health adaptation"[All Fields]) | June 1–15, 2025 | English; 2010–2025 |
| **Web of Science** | TS=("climate change" OR "global warming" OR "environmental change") AND TS=("infectious disease" OR "epidemiology" OR "surveillance" OR "public health adaptation") | June 1–15, 2025 | English; 2010–2025 |
| **Scopus** | TITLE-ABS-KEY("climate change" OR "global warming" OR "environmental change") AND TITLE-ABS-KEY("infectious disease" OR "epidemiology" OR "surveillance" OR "public health adaptation") | June 1–15, 2025 | English; 2010–2025 |

| **Supplementary Table 2. Data Extraction Form Template** | | |
| --- | --- | --- |
| **Variable Name** | **Description** | **Example Entry** |
| **Publication year** | Year the study was published | 2024 |
| **Country or region of study** | Country or region where the study was conducted | United States |
| **Study design** | Type of study design (e.g., modeling, observational) | Modeling study |
| **Climate factor** | Main climate factors reported | Temperature increase |
| **Disease type** | Type of infectious disease studied | Vector-borne disease |
| **Affected population** | Population affected by the disease | General population |
| **Public health intervention type** | Type of intervention or public health measure described | Surveillance system |
| **Study outcome** | Main outcome or finding reported | Increase in disease incidence linked to temperature |
| **Methodological approach** | Method used for analysis | Spatial modeling |
| **Key findings** | Summary of key results or conclusions | Higher dengue risk observed with temperature rise |
